# Menstrual Pattern and Characteristics of One-Rod and Two-Rod Levonorgestrel Implant Users

**DOI:** 10.1155/2021/2904542

**Published:** 2021-03-10

**Authors:** Eka Rusdianto Gunardi, Sulaeman Andrianto Susilo

**Affiliations:** Department of Obstetrics and Gynecology, Faculty of Medicine, University of Indonesia, Jl. Salemba Raya No. 6, Jakarta Pusat 10430, Jakarta, Indonesia

## Abstract

**Introduction:**

The maternal mortality ratio (MMR) in Indonesia reaches 359 per 100,000 live births. The long-acting reversible contraceptive (LARC) method is an effective contraceptive choice for reducing MMR. The contraceptive implant is one of the LARCs that has low usage due to lack of education about the side effects. This study aims to compare the menstrual pattern and characteristics between one-rod and two-rod levonorgestrel implant users.

**Methods:**

A prospective cohort study was performed in patients at Cipto Mangunkusumo Hospital (RSCM) from March 2016 to May 2018. Subject recruitment was done by consecutive sampling. This study was conducted from March 2016 until May 2019. Statistical analysis was performed on the data using the chi-square test to determine the relationship between menstrual pattern and characteristics, and the use of one-rod or two-rod levonorgestrel implants.

**Results:**

A total of 140 subjects participated in the study, comprising 70 (50%) one-rod users and 70 (50%) two-rod users. In the first month, 32.9% one-rod users experienced amenorrhea, 22.9% experienced shortened menstrual period, 30% experienced normal menstrual period, and 14,2 % experienced lengthened menstrual period. In comparison, in the first month, 41.4% two-rod users experienced amenorrhea, 15.7% experienced shortened menstrual period, 32.9% experienced normal menstrual period, and 10% experienced lengthened menstrual period. There was no significant difference in menstrual patterns and characteristics between one-rod and two-rod levonorgestrel implant users.

**Conclusion:**

There was no significant difference in menstrual patterns and characteristics between one-rod and two-rod levonorgestrel implant users. *Implications*. Menstrual patterns and characteristics from levonorgestrel implants user can help clinicians to reduce discontinuation rate from the acceptors. Further research should be conducted to know other side effects aside from menstrual bleeding patterns.

## 1. Introduction

Maternal death is a health problem experienced in both developing countries and developed countries. According to WHO (World Health Organization), maternal death is death that occurs during pregnancy and childbirth until 42 days of termination of pregnancy, from any cause related to or aggravated by the pregnancy or its management but not from accidental or incidental causes [1, 2]. Every day, around 830 women die from preventable diseases related to pregnancy and childbirth. In 2015, the MMR in developing countries was around 239 per 100,000 live births, while in long-acting reversible contraceptive (LARC) method is one of the contraceptive methods that are effective in reducing maternal death [1, 2]. LARCs include the IUD (intrauterine device), levonorgestrel IUD, and progestin implant [3, 4]. Of all the LARCs, implants are the most effective contraception with a failure rate of less than 1% [5–7]. This contraceptive method fits women with poor compliance [8, 9]. After removal of the implant, fertility returns with no side effects [10]. In terms of safety and usage, the implant is safe for women who are breastfeeding [11].

In Indonesia, the family planning program has been proven effective in providing contraceptive services as shown by the increase in the CPR (Contraceptive Prevalence Rate) from 26% in 1970 to 60% in 2002. In 2008–2010 the usage of LARC was relatively fixed. According to the results of the 2010 Mini Survey, the usage of LARC only reached around 11.6%. Based on Indonesia's 2012 demographic health survey, implant contraceptive has few users which can be explained by the lack of information on its side effects [12, 13]. The most commonly reported side effect is irregular menstrual bleeding which often causes users to discontinue implant use [14–17]. Changes in menstrual patterns such as amenorrhea, spotting, and prolonged menstruation are often complained of by implant users [18–21]. Other factors affecting menstrual patterns include breastfeeding and body mass index. All of this needs to be communicated to patients when conducting family planning counselling to increase the number of implant users [8, 22, 23].

These days, the device placed under the skin consists of one rod and two rods. The two-rod implant contains levonorgestrel, while the one-rod implant contains levonorgestrel or etonogestrel (3-ketodesogestrel) [24]. Levonorgestrel implant inhibits sperm transport, affects endometrial development, and partially affects ovulation. Etonogestrel implant works by inhibiting ovulation [24, 25]. The one-rod implant containing levonorgestrel was developed in Indonesia to make it easier for health workers to insert and remove the implant [18].

Previously, there has been no research on the characteristics of menstrual patterns as the side effect of the one-rod implant contraceptive method in Indonesia. At present, the one-rod levonorgestrel implant is a local Indonesian product in phase III clinical trial research phase. Therefore, it is important to compare the characteristics of menstrual patterns between the one-rod and the two-rod implant. Furthermore, we investigated whether there was a relationship between menstrual pattern characteristics in one-rod and two-rod implants with body mass index and breastfeeding.

## 2. Methods

A prospective cohort study was performed in patients at Cipto Mangunkusumo Hospital (RSCM) from March 2016 to May 2018 with the target population mothers with the one-rod and two-rod levonorgestrel implant. Inclusion criteria include complete medical record data. Exclusion criteria include a family history of cancer, use of drugs induced by liver enzymes, use of drugs that interfere with coagulation, high blood pressure, severe hirsutism, and participation in other clinical studies in the last 3 months. The dropout criteria include incomplete menstrual diary, the subject does not return for check-ups or relocates, and the subject passed away. The subjects were given a menstrual book to write their symptoms every month including the characteristics of the bleeding and the amount of the pad which they used. The menstrual book consists of table which can be filled by the subjects each day about their menstrual pattern such as bleeding, spotting, or no bleeding.

Bleeding was defined as any bloody vaginal discharge that required the use of >1 pad or tampon per day. Spotting was defined as any bloody vaginal discharge that required ≤1 pad or tampon per day. A bleeding or spotting episode was defined as ≥1 consecutive day in which bleeding or spotting was recorded, where each episode was bounded by at least 1 bleeding and spotting-free day on either side (note that by this definition 1 bleeding and spot-ting-free day ends an episode). Given a 28-day cycle, a 90-day RP would, therefore, be expected to contain 3.2 menses or bleeding episodes. As per WHO definitions, 3 to 5 bleeding/spotting episodes within a 90-day RP are considered to be a normal frequency of “menses” or bleeding episodes. Amenorrhea was defined as no bleeding or spotting within a 90-day time interval, shortened bleeding was defined as <3 episodes of bleeding/spotting within a 90 days, and lengthened bleeding was defined as ≥1 bleeding episode that began within a 90-day RP and lasted for >14 consecutive days.

The subjects were requested to have their menstrual book checked every 3 months. We did not use the pictorial blood assessment chart because we want to make it easier for the users to write down in the menstrual book; we have already educated the subjects to change their pad if the pad was full. Normal menstrual volume is defined if the amount of bleeding is < 60 ml. Assuming one sanitary pad accommodates 20 ml of blood, we determined a cut-off value of three pads.

In this study, we also identify the relationship between body mass index (BMI) and menstrual pattern characteristics. The BMI was calculated before implant placement. Asian WHO criteria for BMI were used, underweight was defined as BMI < 18.5 kg/m^2^, normal weight was BMI of 18.5–22.9 kg/m^2^, overweight was classified as BMI of 23–24.9 kg/m^2^, obese grade 1 was BMI of 25–29.9 kg/m^2^, and obese grade 2 was BMI ≥ 30 kg/m^2^.

We performed statistical analysis using SPSS version 30 and reported data descriptively using tables.

## 3. Results

A total of 152 subjects participated in the study, with 140 subjects comprising 70 (50%) one-rod users and 70 (50%) two-rod users. Baseline characteristics of subjects receiving one-rod and two-rod implant are presented in Table 1. Twelve subjects were excluded due to cancellation of the research during follow-up (subjects want to have kids). There were no dropouts due to the failure of contraception. This study was double blinded, we had an enveloped which were randomized consisting of one-rod or two-rod levonorgestrel implant.

All variables (i.e., age, parity, BMI, history of abortion, wanting to become pregnant again, history of delivery, breastfeeding, previous contraception used, and duration of insertion) had normal distributions when tested with the Shapiro–Wilk normality test (*p* > 0.05). Age of participants ranged between 20 and 36 years (mean 29 years). There were 51 (72.9%) multipara patients in both one-rod and two-rod users. The BMI ranged equally from underweight to obesity second degree in both one-rod and two-rod users (*p* > 0.05). A history of abortion was reported in 6 (8.6%) one-rod users and 4 (5.7%) two-rod users. More one-rod users than two-rod users wanted to become pregnant again (i.e., 43 (61,4%) versus 33 (47,1%), respectively). Most participants gave vaginal delivery, 61 (81.7%) versus 55 (78.6%) in one-rod users and two-rod users, respectively. Duration of implant insertion was slightly longer in two-rod users compared to one-rod users, that is, 2.97 ± 0.57 minutes versus 2.53 ± 0.73 minutes. There was no difference between one-rod users and two-rod users in previous contraceptive methods used (i.e., pill, implant, injection, IUD, and no history).

Characteristics of menstrual pattern in one-rod users and two-rod users are presented in Tables 2 and 3, respectively. The incidence of amenorrhea in the first month of implant users with one rod and two rods were 23 (23.9%) and 29 (41.4%), respectively, with a slight decrease at 24 months (i.e., 21 (30%) and 20 (27.5%), respectively). The incidence of a shortened period in the first month of implant users with one rod and two rods were 16 (22.9%) and 11 (15.7%), respectively, which reduced drastically at 24 months (i.e., 4 (5.7%) and 3 (4.3%), respectively. The incidence of a lengthened period in the first month of implant users with one rod and two rods were 10 (14.2%) and 7 (10%), respectively, which did not change much at 24 months (i.e., 13 (18.6%) and 7 (10%), respectively).

Characteristics of blood loss in one-rod users and two-rod users are presented in Tables 4 and 5, respectively. In both, one-rod and two-rod users, one-third subjects experienced amenorrhea after one month of implant insertion. Blood loss was evaluated based on the number of sanitary pads used and categorized into less than or equal to three sanitary pads or more than three sanitary pads a day. In the first month after implant insertion, 41 (58.6%) one-rod users and 36 (51.4%) two-rod users used less than or equal to three sanitary pads a day. After 24 months, 40 (57.1%) one-rod users and 42 (60.9%) two-rod users used less than or equal to three sanitary pads a day.

The relationship between BMI and the incidence of amenorrhea is presented in Tables 6 and 7, respectively. There was no statistically significant difference in the incidence of amenorrhea between BMI classes. At one month after implant insertion, the incidence of amenorrhea in one-rod users was 4.3%, 26.1%, 17.4%, 34.8%, and 17.4% in underweight, normal-weight, overweight, obesity first degree, and obesity second degree, respectively. Meanwhile, at one month after implant insertion, the incidence of amenorrhea in two-rod users was 3.4%, 55.2%, 13.8%, 24.1%, and 10.6% in underweight, normal-weight, overweight, obesity first degree, and obesity second degree, respectively. Similar to one month after insertion, after two years of insertion, the incidence of amenorrhea was the highest in obesity first degree (33.3%) for one-rod users and normal BMI (50%) for two-rod use.

The relationship between breastfeeding and the incidence of amenorrhea is presented in Tables 8 and 9, respectively. At one month, the incidence of amenorrhea and not amenorrhea, in breastfeeding one-rod users, were 9 (30%) and 21 (70%), respectively. At 6 months, the incidence of amenorrhea and not amenorrhea, in breastfeeding one-rod users, were 12 (40%) and 18 (60%), respectively. There was no statistically significant difference in menstrual patterns in one-rod users who breastfeed and not from months 1–6. In comparison, there was a statistically significant difference in menstrual patterns in two-rod users who breastfeed and not from months 1–6. At one month, the incidence of amenorrhea and not amenorrhea, in breastfeeding two-rod users, were 16 (59%) and 11 (30%), respectively (*p*=0.016). At 6 months, the incidence of amenorrhea and not amenorrhea, in breastfeeding two-rod users, were 17 (62%) and 10 (37%), respectively (*p*=0.036).

## 4. Discussion

Levonorgestrel implant usage provides good cervical mucus viscosity until the third month of implant usage, giving effectiveness of up to 96.7% [18]. The National Population and Family Planning Board reported the implant as having the smallest failure rate of 1% among long-acting reversible contraceptives (LARC) [12].

Levonorgestrel concentration reaches its peak after 24–72 hours after implant insertion and decreases in the first week. The average concentration is attained in the first month which will decrease gradually during three years of usage. Gunardi et al. showed that there was no serum level difference in levonorgestrel between one-rod and two-rod users. Effectiveness of implant has depended on a minimum serum level of levonorgestrel of 200 pg/mL with no difference in the effectiveness between one-rod and two-rod implant [18].

Multipara women represented more than two-thirds (72.9%) of subjects in both one-rod and two-rod users. In comparison, Kavanaugh et al. reported a study in which long-acting reversible contraceptives were greater in primipara women [26]. This difference could be due to the lack of encouragement to use contraception which increased the number of children of women sampled in this study.

The body mass index (BMI) profile of subjects in this study did not differ much with the study by Bhuva. et al with most subjects having a normal or overweight BMI. One reason for this suggested Bhuva et al. could be that women with excess weight avoid using hormonal contraception because of the fear of gaining weight [27].

Forty-three (61.4%) one-rod users did not want to have children again, while 27 (38.6%) users still wanted to have children. Thirty-three (47.1%) two-rod users did not want to have children again, while 37 (52.9%) users still wanted to have children. This finding is consistent with a data registry in the United States in 2009–2012, which states that the use of long-acting reversible contraceptives (LARC) is more widely used by those who no longer want to have children. This study also states that there is a statistically significant correlation between the use of LARC with the desire to have more children [26].

Most subjects, 31 (45.7%) one-rod users and 31 (44.3%) two-rod users, used injection contraceptive before using the implant. The preferences of contraceptive users according to a registry conducted in the United States by the National Survey of Family Growth in 2009–2012 were the IUD (both hormonal or nonhormonal) followed by the implant [26].

Changes in menstrual patterns are the most frequent complaint of implant users. The mechanism of this change is a fluctuating increase in ovary estradiol and a continuous exposure of progesterone to the endometrial gland, stroma, and vascular. This causes disturbances in angiogenesis that become brittle and thin, lack of peripheral cells, defects of the basal membrane, impaired migration of leukocytes, and disruption in the distribution of metalloprotease matrix release [28, 29].

We found no differences in menstrual patterns and characteristics between one-rod and two-rod levonorgestrel implant users. It is coherent with one of the clinical trials from Gunardi et al. that shows no significant difference between the menstrual pattern, effectiveness, safety, and time in levonorgestrel levels in 24th month between one-rod and two-rod implant [30]. Amenorrhea was the most common menstrual pattern found in this study. This is consistent with the study of Sivin et al. in which the incidence of amenorrhea increased five times more in subjects with levonorgestrel implants [31]. We found no difference in menstrual patterns and characteristics between one-rod and two-rod levonorgestrel implant users.

We found no statistically significant difference in blood loss between one-rod or two-rod users, with more than 80% using less than or equal to three sanitary towels. This is in line with the research of Fraser and Christine Roke which reported less blood loss and amenorrhea [32, 33]. Assessment based on the number of pads is not an appropriate parameter to assess the volume of blood loss due to differences in perception of the subjects and no specific provisions on the type of pads used. Subjective assessment is not an accurate step to diagnose the condition. However, this assessment can reduce medical intervention is cost-saving. Assessment using a menstrual pictogram is an easy, accurate, and objective way to estimate the amount of bleeding [34].

Two variables that will also affect menstrual patterns are body mass index and breastfeeding. Bleeding pattern was monitored from the first month to 24 months. We found no statistically significant difference in the incidence of amenorrhea between BMI classes. This is contrary to the study of Sivin et al. which showed that there was a correlation between body weight in women using levonorgestrel implants and changes in menstrual patterns. Women with less body weight had fewer menstrual episodes and longer bleeding-free interval, resulting in a greater proportion of women with oligomenorrhea or amenorrhea. More weight was associated with more bleeding days and shorter intervals between bleeding episodes. This relationship seems to exist within and between geographical regions. Studies in countries with a lower average of weight show a high proportion of women who experience amenorrhea with levonorgestrel implants. The response to amenorrhea due to implant use varies considerably between individuals and cultures [31].

We found a statistically significant difference in menstrual patterns in two-rod users who breastfeed and not from months 1–6. Diaz et al. reported that bleeding irregularity rarely occurs during breastfeeding. Levonorgestrel implant users experience a period of lactation amenorrhea 4 to 5 months longer than T-Cu users at the beginning of contraception use. More than half of the women in each contraceptive group reported bleeding in the first month after starting treatment (72% and 85% of Norplant and T-Cu users, respectively, compared to 26% of women who did not use birth control in the same period). Prolonged or frequent bleeding rarely occurred in the first 12 months after starting treatment. However, the proportion of bleeding that lasted more than 10 days was significant in the levonorgestrel implant group (7.0% of 628 observed bleeding) compared to the T-Cu group (3.0% of 1169 bleeding) and in women who did not use birth control (0.6% of 479 bleeding) [35]. In theory, the process of breastfeeding also tends to lead to amenorrhea. However, changes in menstrual patterns in each subject are different, so it is necessary to educate about the possibility of change in menstrual patterns in subjects who are breastfeeding.

## 5. Conclusion

There was no significant difference in menstrual patterns and characteristics between one-rod and two-rod levonorgestrel implant users. It is consistent with other comparative studies which show levonorgestrel level was the same in 24-month follow-up with no difference in symptoms among two implants. Characteristics of bleeding most often start with amenorrhea in the first month of implant insertion which gradually improves until the second year. The pattern of bleeding observed from the first month to the second year showed that the bleeding pattern was improving during the duration of two years. There was no statistically significant difference in the incidence of amenorrhea between BMI classes in both one-rod and two-rod users. At 6 months after insertion in two-rod users, the incidence of amenorrhea was significantly higher in breastfeeding women (60%). This is in contrast to the incidence of amenorrhea in one-rod users which showed no significant relationship with breastfeeding. However, the development of menstrual patterns in both one-rod and two-rod users showed no change.

## Figures and Tables

**Table 1:** Baseline characteristics of subjects receiving one-rod and two-rods implant.

Variable	One-rod implant (*n* = 70)	Two-rod implant (*n* = 70)
*Age (mean* *+* *SD)*	28.4 ± 4.2	28.6 ± 4.1

*Parity*		
Primipara	19 (27.1%)	19 (27.1%)
Multipara	51 (72.9%)	51 (72.9%)

*BMI*		
Underweight	8 (11.4%)	7 (10%)
Normal	20 (28.6%)	29 (41.4%)
Overweight	13 (18.6%)	10 (14.3%)
Obesity first degree	20 (28.6%)	16 (22.9%)
Obesity second degree	9 (12.9%)	8 (11.4%)

*History of abortion*		
Yes	6 (8.6%)	4 (5.7%)
No	64 (91.4%)	66 (94.3%)

*Wanting to become pregnant again*		
Yes	27 (38.6%)	37 (52.9%)
No	43 (61.4%)	33 (47.1%)

*History of delivery*		
Vaginal birth	61 (87.1%)	55 (78.6%)
Caesarean section	9 (12.9%)	15 (21.4%)

*Breastfeeding*		
Yes	30 (42.9%)	27 (38.6%)
No	40 (57.1%)	43 (61.4%)

*Previous contraception used*		
Pill	13 (18.6%)	8 (11.4%)
Implant	6 (8.6%)	11 (15.7%)
Injection	32 (45.7%)	31 (44.3%)
IUD	3 (4.3%)	3 (4.3%)
No history	16 (22.9%)	17 (24.3%)

*Duration of insertion (minutes)*	2.53 ± 0.73	2.97 ± 0.57

**Table 2:** Characteristics of menstrual pattern in one-rod users.

Month	Menstrual pattern				
	Amenorrhea	Shortened	Normal	Lengthened	*p*
1	23 (32.9%)	16 (22.9%)	21 (30%)	10 (14.2%)	0.52
3	25 (35.7%)	17 (24.3%)	20 (28.6%)	8 (11.4%)	0.29
6	31 (44.3%)	20 (28.6%)	12 (17.1%)	7 (10%)	0.77
12	29 (41.4%)	11 (15.7%)	24 (34.3%)	6 (8.6%)	0.94
18	24 (34.3%)	14 (20%)	21 (30%)	11 (15.7%)	0.54
24	21 (30%)	4 (5.7%)	32 (45.7%)	13 (18.6%)	0.40

**Table 3:** Characteristics of menstrual pattern in two-rod users.

Month	Menstrual pattern				
	Amenorrhea	Shortened	Normal	Lengthened	*p*
1	29 (41.4%)	11 (15.7%)	23 (32.9%)	7 (10 %)	0.52
3	32 (45.7%)	11 (15.7%)	23 (32.9%)	4 (5.7%)	0.29
6	33 (47.1%)	15 (21.4%)	15 (21.4%)	7 (10.1%)	0.77
12	30 (42.9%)	13 (18.6%)	21 (30%)	6 (8.5%)	0.94
18	28 (40%)	12 (17.1%)	24 (34.3%)	6 (8.6%)	0.54
24	20 (27.5%)	3 (4.3%)	40 (58%)	7 (10.2%)	0.40

**Table 4:** Characteristics of blood loss in one-rod users.

Month	Blood loss			
	Amenorrhea	≤3 sanitary pads	>3 sanitary pads	*p*
1	23 (32.9%)	41 (58.6%)	6 (8.5%)	0.67
3	25 (35.7%)	38 (54.3%)	7 (10%)	0.32
6	31 (44.3%)	37 (52.9%)	2 (2.8%)	0.94
12	29 (41.4%)	29 (41.4%)	12 (17.2%)	0.93
18	24 (34.3%)	40 (57.1%)	6 (8.6%)	0.69
24	21 (30%)	40 (57.1%)	9 (12.9%)	0.9

**Table 5:** Characteristics of blood loss in two-rod users.

Month	Blood loss			
	Amenorrhea	≤3 sanitary pads	>3 sanitary pads	*p*
1	29 (41.4%)	36 (51.4%)	5 (7.2%)	0.67
3	32 (45.7%)	33 (47.1%)	5 (7.2%)	0.32
6	33 (47.1%)	35 (50%)	2 (2.9%)	0.94
12	30 (42.9%)	27 (38.6%)	13 (18.5%)	0.93
18	28 (40%)	35 (50%)	7 (10%)	0.69
24	20 (27.5%)	42 (60.9%)	8 (11.6%)	0.9

**Table 6:** The relationship between BMI and amenorrhea in one-rod users.

Month	Menstrual pattern	BMI classification					
		Underweight	Normal	Overweight	Obesity first degree	Obesity second degree	*p*
1	Amenorrhea	1 (4.3%)	6 (26.1%)	4 (17.4%)	8 (34.8%)	4 (17.4%)	0.625
	No amenorrhea	7 (14.9%)	14 (29.8%)	9 (19.1%)	12 (25.5%)	5 (10.6%)	

12	Amenorrhea	2 (6.9%)	8 (27.6%)	7 (24.1%)	9 (31%)	3 (10.3%)	0.721
	No amenorrhea	6 (14.6%)	12 (29.3%)	6 (14.6%)	11 (26.8%)	6 (14.6%)	

24	Amenorrhea	3 (14.3%)	4 (19%)	5 (23.8%)	7 (33.3%)	2 (9.5%)	0.716
	No amenorrhea	5 (10.2%)	16 (32.7%)	8 (16.3%)	13 (26.5%)	7 (14.3%)	

**Table 7:** The relationship between BMI and amenorrhea in two-rod users.

Month	Menstrual pattern	BMI classification					
		Underweight	Normal	Overweight	Obesity first degree	Obesity second degree	*p*
1	Amenorrhea	1 (3.4%)	16 (55.2%)	4 (13.8%)	7 (24.1%)	1 (3.4%)	0.126
	No amenorrhea	6 (14.6%)	13 (31.7%)	6 (14.6%)	9 (22%)	7 (17.1%)	

12	Amenorrhea	3 (10%)	16 (53.3%)	5 (16.7%)	5 (16.7%)	1 (3.3%)	0.207
	No amenorrhea	4 (10%)	13 (32.5%)	5 (12.5%)	11 (27.5%)	7 (17.5%)	

24	Amenorrhea	0 (0%)	10 (50%)	3 (15%)	6 (30%)	1 (5%)	0.293
	No amenorrhea	7 (14%)	19 (38%)	7 (14%)	10 (20%)	7 (14%)	

**Table 8:** The relationship between breastfeeding and amenorrhea in one-rod users.

Month	Menstrual pattern	Breastfeeding		
		No	Yes	*p*
1	Amenorrhea	14 (35%)	9 (30%)	0.659
	Not amenorrhea	26 (65%)	21 (70%)	

3	Amenorrhea	13 (32.5%)	12 (40%)	0.517
	Not amenorrhea	27 (67.5%)	18 (60%)	

6	Amenorrhea	19 (47.5%)	12 (40%)	0.532
	Not amenorrhea	21 (52.5%)	18 (60%)	

**Table 9:** The relationship between breastfeeding and amenorrhea in two-rod users.

Month	Menstrual pattern	Breastfeeding		
		No	Yes	*p*
1	Amenorrhea	13 (30%)	16 (59%)	0.016^*∗*^
	Not amenorrhea	30 (70%)	11 (41%)	

3	Amenorrhea	15 (35%)	17 (62%)	0.022^*∗*^
	Not amenorrhea	28 (65%)	10 (37%)	

6	Amenorrhea	16 (38%)	17 (62%)	0.036^*∗*^
	Not amenorrhea	27 (62%)	10 (37%)	

## Data Availability

The datasets used in the study are available from the corresponding author upon request.
